# Susceptibility of Impact Damage to Whole Apples Packaged Inside Molded Fiber and Expanded Polystyrene Trays

**DOI:** 10.3390/foods10091980

**Published:** 2021-08-25

**Authors:** Kyle Dunno, Isabel Stoeckley, Matthew Hofmeister

**Affiliations:** Department of Packaging Science, Rochester Institute of Technology, 78 Lomb Memorial Drive, Rochester, NY 14623, USA; irs2530@g.rit.edu (I.S.); mxh6953@g.rit.edu (M.H.)

**Keywords:** bruise susceptibility, apples, mechanical shock, transportation, molded fiber, expanded polystyrene

## Abstract

Postharvest damage, leading to loss and waste, continues to be a significant problem in the fresh produce industry. Trays, designed to reduce fruit-to-fruit contact, are utilized by the apple industry to minimize bruising of whole apples. During distribution, packaged apples are subjected to various supply chain hazards, which may lead to bruising damage. Currently, molded fiber (MF) and expanded polystyrene (EPS) trays transport whole apples from the packhouse to the retail outlet. Mechanical shock, by free-fall drop method, was used to evaluate the performance differences between the two trays and quantify the bruising characteristics of the apples. Results showed that the EPS trays provided better shock protection to the apple as compared to the MF tray, reducing the impact acceleration by more than 70%. Additionally, the bruise susceptibility was 40% less for the apples packaged inside the EPS trays, regardless of drop height. However, apples packaged in the middle layer trays were most susceptible to bruising damage, regardless of tray type.

## 1. Introduction

Fresh produce travels through a demanding food supply chain to reach the consumer. During this journey, products are exposed to a variety of supply chain hazards, such as mechanical shock, vibration, and compression. In apples, these hazards can result in bruising to the fruit, altering their quality and perceived deterioration by consumers, especially during bulk display at retail [1,2,3,4,5,6]. In today’s market, consumers demand fresh produce to be free from visible defects such as bruising. Excessive defects in the apple will deter consumers, resulting in them selecting other items causing product shrinkage [4]. Although the bruise size is a function of the mechanical properties of the apple flesh, a visible bruise greater than 100 mm^2^ will typically result in the apple being discarded as waste [7,8]. Peggie [9] reported that approximately 8–10% of apples harvested were discarded mainly due to bruise damage. However, Lewis et al. [4] reported data from apple distributors that indicated apple waste could be 50% or higher due to bruising. As a result, the product’s visual appearance is critical to the purchasing instincts of the consumer.

Packaging performs a variety of functions, one of which is to protect the product during transport. For apples, the type of packaging system employed is dependent on its position in the postharvest supply chain. For example, bulk bins are used during post-harvesting to move the apples from the grower to the packhouse, while apples traveling to the retail outlet are packaged inside corrugated containers containing bags, pouches, or trays of apples. Unfortunately, although a wide range of packaging formats are available, most of them are not designed to adequately protect the apples during transit, resulting in bruise damage still being a frequent quality problem for growlers and retailers [1].

Limiting apple-to-apple contact during transport is desirable as this minimizes the opportunity of the fruit to bruise. To accomplish this, apples traveling to retail are often packaged into trays with individual cells. The two predominant tray materials utilized by the apple industry are molded fiber (MF) and expanded polystyrene (EPS). MF, produced from pulp slurry, can be appealing due to its end-of-life impact but is susceptible to moisture gain and swelling, which could be a deterrent for some applications. EPS is a closed-cell foam consisting of 98% air, making it incredibly lightweight while also providing excellent energy absorption and dissipation during impacts. However, in recent years there have been restrictions put in place regarding the use of EPS due to its environmental impact including legislation passed by local and state governments [10]. Food losses and waste (FLW) are globally becoming a top priority in food management in order to increase food security, while also striving to reduce environmental impacts [11]. Therefore, it is imperative to understand not only the environmental impact of the package material, but also understand its influence on food loss. Additional packaging solutions exist for the transport of fresh fruit, such as corrugated sleeves, mesh netting, and stand-up pouches and bags, but these solutions are not commercially used in the transport of apples. Although both MF and EPS have been used extensively by the apple industry to move whole apples to retail, limited data exists comparing these two materials to understand which tray provides better protection against bruising.

Previous research has evaluated the influence of packaging materials on the bruising characteristics of whole apples undergoing transportation simulations, but the vast majority of research available examines impacting the fruit using a pendulum method [5,6,12,13,14,15]. Singh et al. [16] and Singh and Xu [17] examined different packaging materials, including MF and EPS, focused on the damage resulting from laboratory vibration simulations. The results, however, indicated the fruit packaged inside the EPS trays had less damage than those inside the MF trays. Fadiji et al. [1] reported that apples were more susceptible to bruising when packaged inside plastic bags as compared to trays during multiple impacts. By placing the apples inside the individual cells, the fruit-to-fruit impact was reduced [18]. Batt et al. [19] investigated the performance of MF and EPS tray types during simulated transport conditions and noted there were no significant differences in apple bruise frequency or size. However, this project examined only one impact from 61 cm, unlike Fadiji et al. [1], which examined apple bruising using multiple drops from shorter drop heights which are more common for fruit packaged at this stage in the supply chain.

Numerous studies focusing on the design of the ventilated corrugated container for apples and other fruits have been published [20,21,22,23]; however, little is known about the performance of the fruit trays designed to prevent fruit damage. The objective of this research was to investigate the bruise susceptibility of whole apples during mechanical shock inside ventilated corrugated containers when packaged using either MF or EPS trays, including the bruise frequency and impact acceleration experienced by the fruit.

## 2. Materials and Methods

### 2.1. Apple Variety 

‘Minneiska’ (SweeTango^®^) apples packaged using two different tray designs (MF and EPS) were acquired during commercial harvest, in September, from a packhouse in Wolcott, NY, USA. Fruit of uniform size and maturity based on color, firmness, and free from physical defects were used for the experiments. The mean diameter of the apple was 74.4 ± 8.8 mm. The average mass of the apple was 197 ± 25 g. The packaged apples were stored at refrigerated conditions (4 °C) for at least 48 h before the experiment. 

### 2.2. Packaging Materials

**Figure 1:**
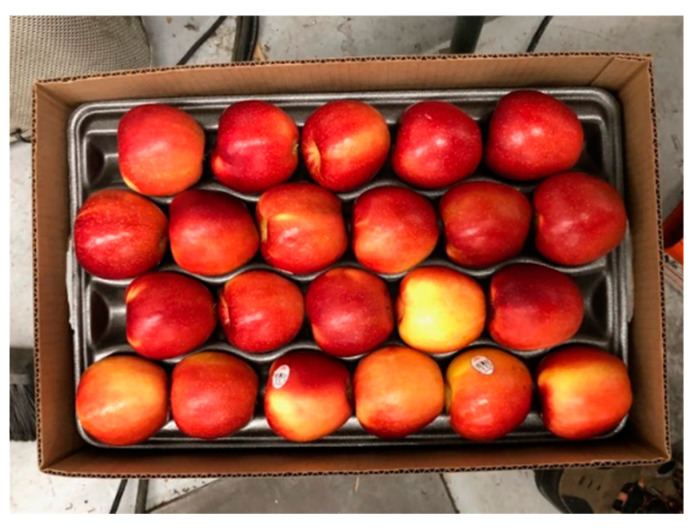
Arrangement of apples in molded pockets prior to testing.

Selected for this study were two interior trays types used for whole apple transport, molded fiber (MF) and expanded polystyrene (EPS). The trays used for this study were standard apple trays designed to hold 88 apples per ventilated corrugated container. Each tray held 22 apples, and there are four trays of apples in each container. The trays were numbered sequentially as Tray 1–4 from bottom to top, starting with Tray 1 located at the bottom of the container. The outer dimensions of the ventilated corrugated container were 514 mm × 327 mm × 635 mm. The final mass of the filled corrugated containers with whole apples was 18.81 ± 0.05 kg and 18.31 ± 0.17 kg for the containers with MF and EPS trays, respectively. The apples were carefully inspected and arranged in the trays with the flower stalk axis horizontal and running parallel with the molded pockets of the trays (Figure 1). 

### 2.3. Free-Fall Drop Test

**Figure 2:**
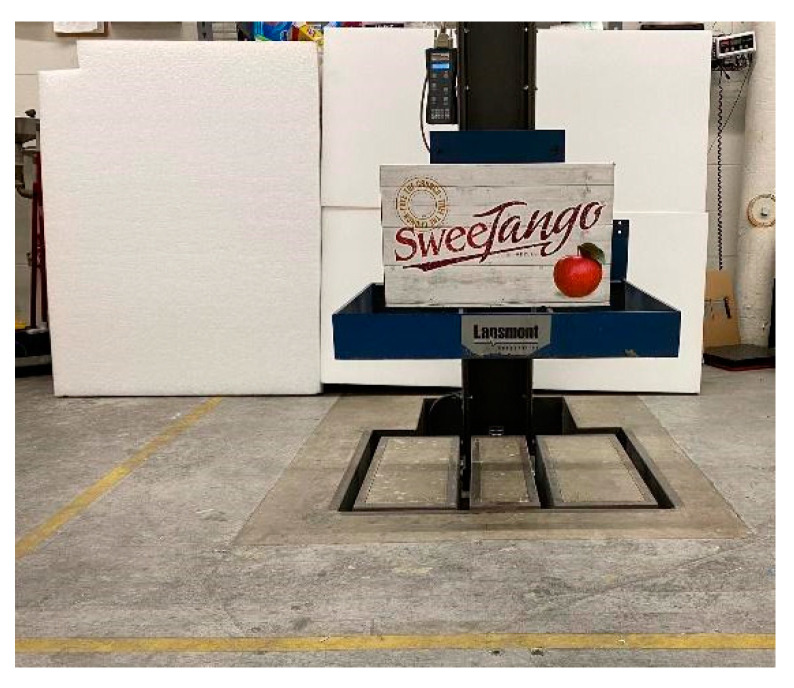
Packaged product on the Lansmont PDT 227.

A Lansmont Model PDT 227 Precision Drop Tester (Lansmont Corporation, Monterey, CA, USA) was used to perform the free-fall drop events (Figure 2). Impact bruises were produced by dropping each corrugated container five times from a predetermined drop height onto the concrete floor surface of the drop test equipment. In this study, the packages were dropped onto the bottom panel of the package from two drop heights, 30 cm and 50 cm. The testing was performed in duplicate for each package configuration at the two different drop heights. 

To record the impact acceleration of the apple from the free-fall drop event, a model HT356B21 triaxial accelerometer (PCB Piezotronics, Inc., Depew, NY, USA) was attached to the apple located in the corner cell of the top tray. The sensor was placed in this position to ensure it would not shear off during the impacts with surrounding apples. The Lansmont Test Partner 3 (Lansmont Corporation, Monterey, CA, USA) processed the signal events and the resultant acceleration from each impact recorded.

### 2.4. Bruise Analysis 

**Figure 3:**
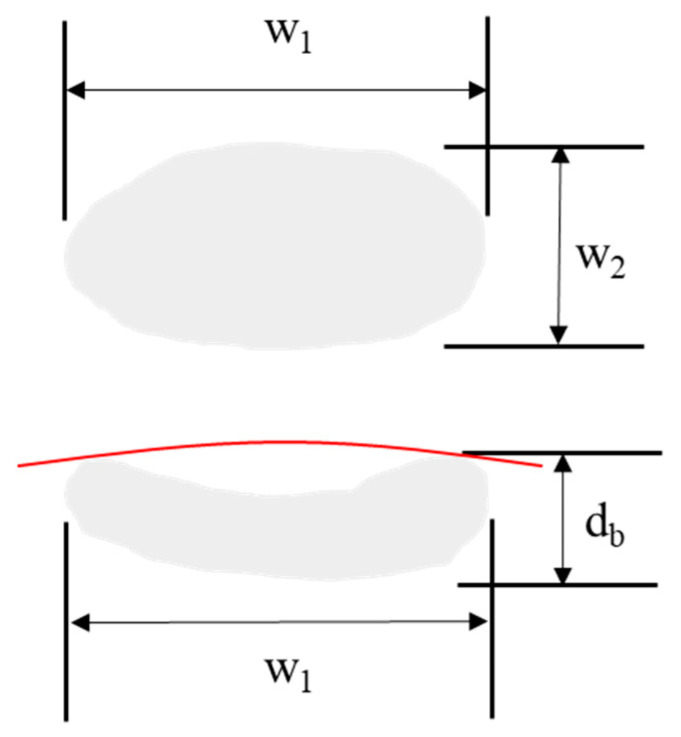
Elliptical bruise thickness method used for BA and BV.

To allow for the full development of bruising sustained during the mechanical shock testing, the apples were kept at ambient laboratory conditions after drop testing for a period of 24 h, prior to inspection and analysis. The total number of bruises per apple was recorded for each tray to determine the frequency of occurrence and distribution of bruising inside the container. The bruise area (BA) and bruise volume (BV) were measured for each apple after testing. The bruise dimensions were measured using digital calipers (±0.01 mm). The BA was measured using the major and minor bruise width, and the BV was calculated by measuring the depth of the bruise by cutting the fruit perpendicularly along the major bruise width (Figure 3). BA and BV were quantified using an assumed elliptical bruise shape [24,25,26] using Equations (1) and (2).

(1) $$BA=\frac{\pi }{4}w_{1}w_{2}$$

(2) $$BV=\pi \frac{d_{b}}{24}(3w_{1}w_{2}+4d_{b}^{2})$$

where *w*_1_ is the bruise width along the major axis (mm), *w*_2_ is the bruise width along the minor axis (mm), and *d_b_* is the depth of the bruise (mm)

The bruise susceptibility (BS) was computed as the ratio of the *BV* to the impact energy (*IE*), as shown in Equation (3) [2,26]. Table 1 and Equation (4) display how *IE* was calculated for each tray type.

(3) $$BS=\frac{BV}{IE}$$

where *BV* is the bruise volume (mm^3^) and *IE* is the impact energy (J).

(4) $$IE=m_{i}gh_{d}$$

where *m_i_* is the mass of the falling object (kg), *g* is the acceleration due to gravity (m/s^2^), and *h_d_* is the drop height (m).

### 2.5. Tray Damage Assessment

At the completion of each drop test, the trays were visually inspected for damage sustained during the mechanical shock testing. The condition of the tray was graded using a qualitative assessment based on the level of damage (i.e., tearing and cracking). The scale used to grade the condition after the drop test was poor (severe damage), fair (moderate damage), or good (minor damage) based on the damage level.

### 2.6. Statistical Analysis

The data were statistically analyzed using a one-way analysis of variance (ANOVA) at a 95% confidence level to compare the results collected during this experiment. Statistical differences between treatments were noted where *p* < 0.05. The experiment was performed in duplicate for the two packaging materials at the different heights. All statistical analyses and resulting graphical outputs were performed using Minitab (v. 18 Minitab, LLC, State College, PA, USA).

## 3. Results and Discussion

### 3.1. Bruise Analysis

**Figure 4:**
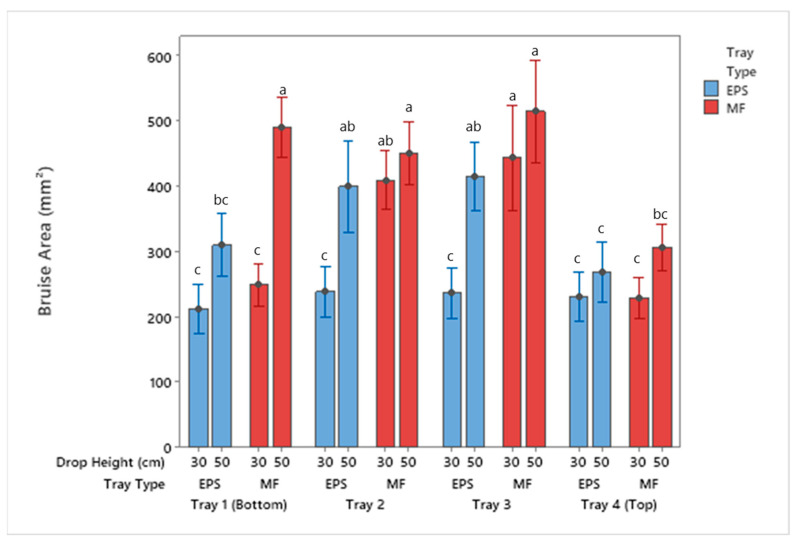
Bruise area of apples by tray location packaged using MF and EPS trays from 30 and 50 cm drop height (mean ± SD, *n* = 2). Different letters indicate statistically significant differences at *p* < 0.05. Bars with no common letters are significantly different (*p* < 0.05).

**Figure 5:**
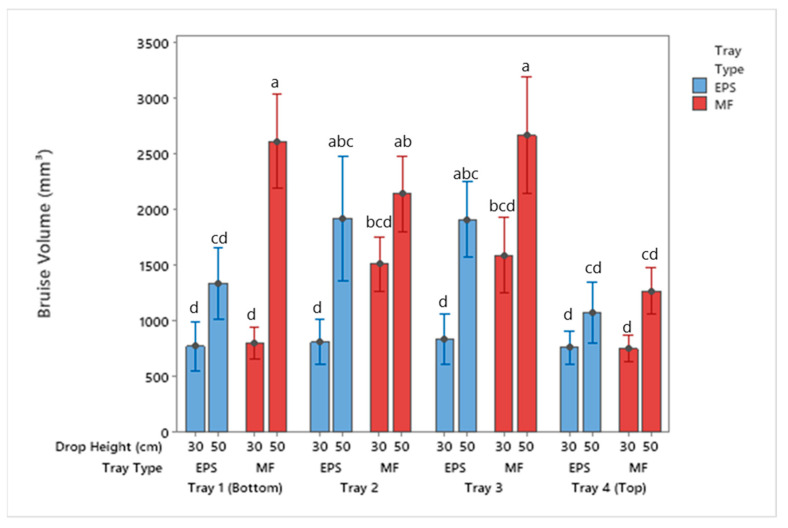
Bruise volume of apples by tray location packaged using MF and EPS trays from 30 and 50 cm drop height (mean ± SD, *n* = 2). Different letters indicate statistically significant differences at *p* < 0.05. Bars with no common letters are significantly different (*p* < 0.05).

The results in Figure 4 and Figure 5 show the total apple bruise area and volume for each tray material after completion of drop testing. As the drop height increased from 30 cm to 50 cm, the bruise sizes increased in both EPS and MF. As demonstrated by Lu et al. [25] and T. Fadiji et al. [1], the bruise area and volume increased due to drop height and number of drops. For a bruise to form, the apples absorb kinetic energy that is not dissipated by the packaging material [5,27,28,29]. At both test drop heights, the apples in the MF trays experienced more damage and bruising. Both tray systems prevent direct apple-to-apple contact, but the protective nature of the EPS material plays a crucial role in minimizing bruising. The lower bruise damage results from the EPS trays absorbing more kinetic energy than the MF trays from the same drop height. The amount of bruising observed on the apples is dependent on the amount of energy absorbed by the packaging in the apple supply chain. The amount of bruising directly affects the quality of the apple fruit and the consumers purchasing behavior [5,26,30,31].

There was an increase in bruise area and volume for both the MF and EPS when comparing the 30 cm to 50 cm drop height results. The differences in the bruise area were not significant for Trays 2–4 but were significantly different for Tray 1 for the MF trays. The bruise area for apples packaged with EPS trays was not significantly different for Trays 1 and 4, but was for Trays 2 and 3. Furthermore, when comparing these results, no significance was reported between the MF Trays 2 and 3 dropped from 30 cm and EPS Trays 2 and 3 dropped from 50 cm. This indicates the apples dropped from 50 cm with EPS trays had similar damage to apples dropped from 30 cm inside MF trays.

Evaluating the results from the 30 cm drop height, no statistical differences in tray location between apples packaged with EPS trays were observed. The 30 cm drop resulted in impact energies low enough to be within the cushion material’s working length, resulting in a similar performance of the tray throughout all tests. For the 30 cm drop height, all of the bruise areas were below 240 mm^2^ for the EPS trays. Regarding the MF tray locations, there were significant differences in bruise area and volume for Trays 2 and 3. The bottom and top trays (Trays 1 and 4) were aided in protection by the corrugated container, likely reducing the impact energy on apples in those locations.

Statistical differences were noted for the package system dropped from a height of 50 cm for both the EPS and MF tray types. Tray 4 (top tray) had the smallest bruise area compared to apples located in other tray locations for both tray types. Apples packaged using MF saw significantly greater damage in Trays 1–3 as compared to Tray 4. For apples inside EPS trays, the two middle layers were significantly different than Trays 1 and 4. These results are consistent with Fadiji et al. [1], who noted apples packaged in the middle trays of an MK4 corrugated container had more bruising damage than those packaged in the bottom and top trays.

**Figure 6:**
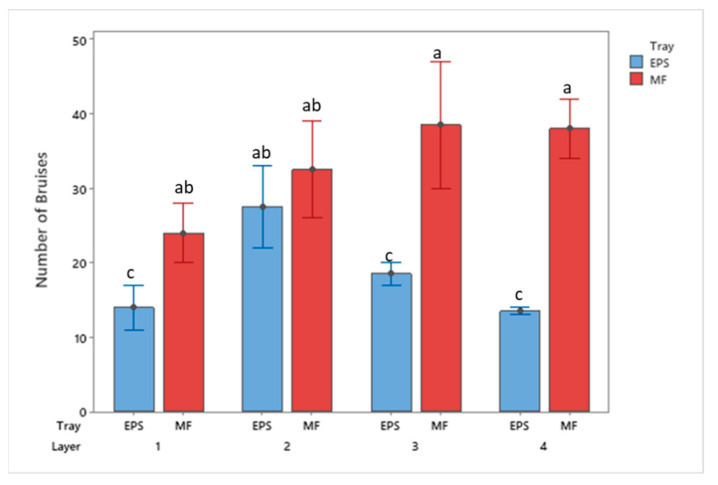
Bruise frequency of apples by tray location packaged using MF and EPS trays from 30 cm drop height (mean ± SD, *n* = 2). Different letters indicate statistically significant differences at *p* < 0.05. Bars with no common letters are significantly different (*p* < 0.05).

**Figure 7:**
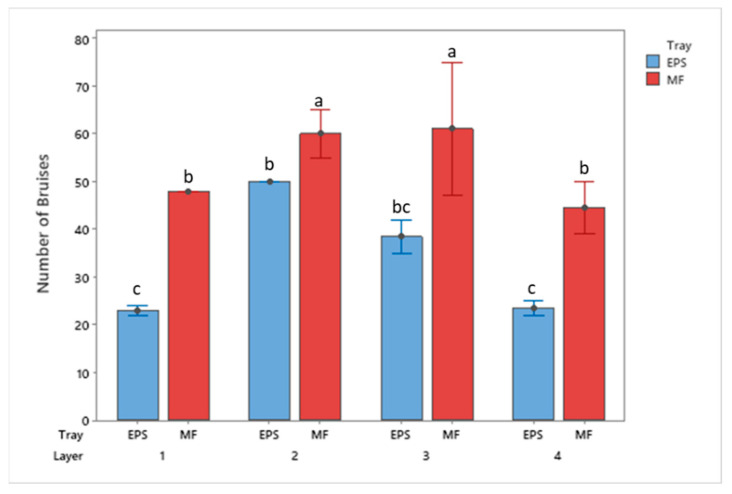
Bruise frequency of apples by tray location packaged using MF and EPS trays from 50 cm drop height (mean ± SD, *n* = 2). Different letters indicate statistically significant differences at *p* < 0.05. Bars with no common letters are significantly different (*p* < 0.05).

Additionally, the frequency, or occurrence, of bruising was observed during the analysis. Figure 6 and Figure 7 display bruise frequency based on the drop height and tray material. Results from the 30 cm and 50 cm drops show EPS trays reported a lower total count of bruises than the MF trays. The MF trays from 30 cm show an increasing trend in the total number of bruising from the bottom to top trays, whereas the remaining treatments have the greatest number of bruises occurring to the middle trays.

### 3.2. Bruise Susceptibility

**Figure 8:**
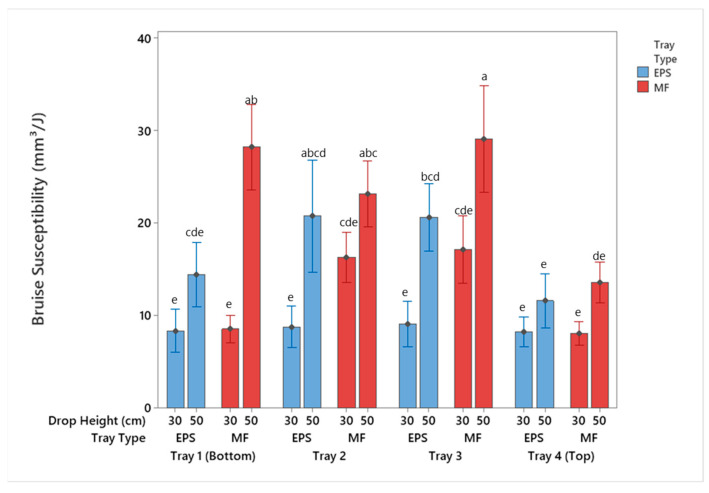
Bruise susceptibility of apples by tray location packaged using MF and EPS trays from 30 and 50 cm drop height (mean ± SD, *n* = 2). Different letters indicate statistically significant differences at *p* < 0.05. Bars with no common letters are significantly different (*p* < 0.05).

Table 1 displays the impact energy associated with each package configuration based on the mass of the package system and the drop height. The impact energies at each of the drop heights were effectively the same, noting the difference being the lighter mass of the EPS tray compared to the MF tray. Although the impact energies of the two package systems were similar, the bruise susceptibility, in terms of the ratio of the bruise volume to the impact energy [7], was greater for apples packaged using MF trays (Figure 8). These results indicate the EPS trays were able to absorb more of the impact energy, reducing the amount of energy transferred to the apple during the impact event. Although the MF tray reduces the lateral movement of the apples, it provides minimal energy absorption during vertical impacts, resulting in the apples being exposed to greater impact energy and subsequent bruising. The apple package system absorbs the energy through stretching of the trays, container sidewall buckling, and compression between the apple contact surfaces [28,32]. The most significant difference in the two package systems evaluated during this study was the compression of the apple contact surfaces. The EPS trays were able to reduce the compression between apples better than the MF trays.

**Figure 9:**
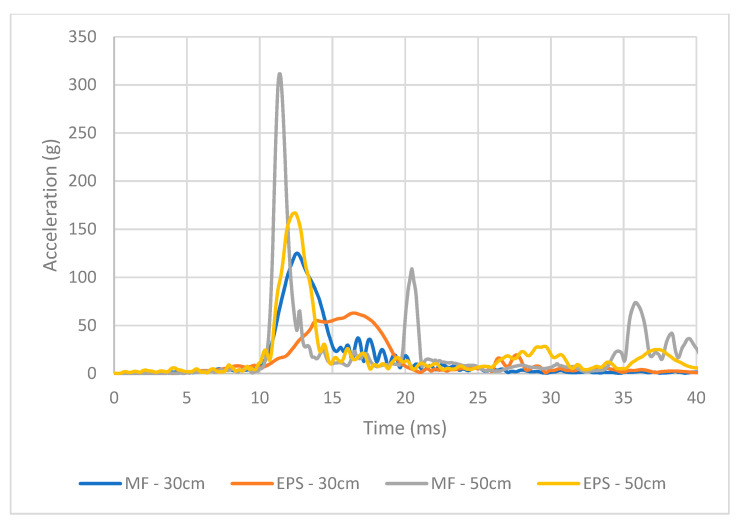
Example of acceleration vs. time curve during impact response from each of the experimental treatments.

**Figure 10:**
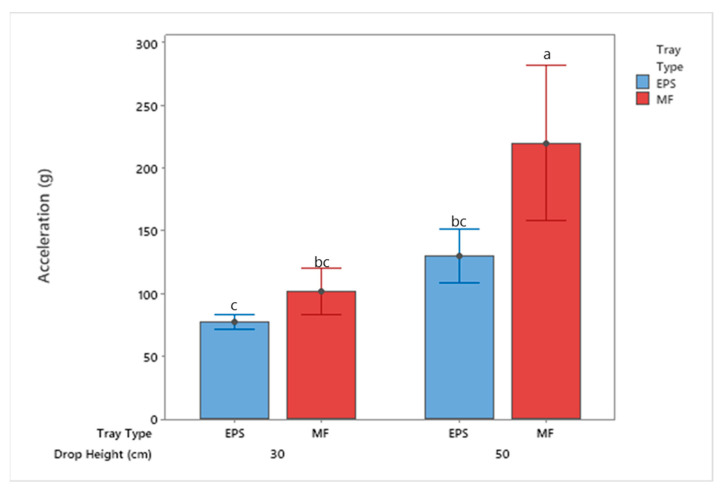
Impact acceleration from the free-fall drop events of apples by tray location packaged using MF and EPS trays from 30 and 50 cm drop height (mean ± SD, *n* = 2). Different letters indicate statistically significant differences at *p* < 0.05. Bars with no common letters are significantly different (*p* < 0.05).

To compare the protective cushion properties of the two trays, the resultant acceleration was recorded for each free-fall event. Figure 9 displays an example acceleration versus time curve from the individual impacts showing the apples’ response to the free-fall impact. The impact accelerations were averaged for comparison for each treatment type (drop height and material type). As shown in Figure 10, the average acceleration of the apples packaged with the EPS tray was significantly less than that of the MF tray for both the 30 cm and 50 cm drops. The impact duration of the apples in the EPS were twice as long on average compared to MF trays. Comparing the trays from the 30 cm drop tests, the acceleration levels experienced by the apples increased by 30% for those packaged with MF trays. Additionally, examining the results from the 50 cm drop tests, the acceleration levels increased by 71% for apples packaged inside the MF as compared to those of the apples packaged inside the EPS trays. These results indicate that the EPS trays could absorb the impact energy from the event, resulting in less bruising as noted in previous sections.

### 3.3. Tray Damage

**Figure 11:**
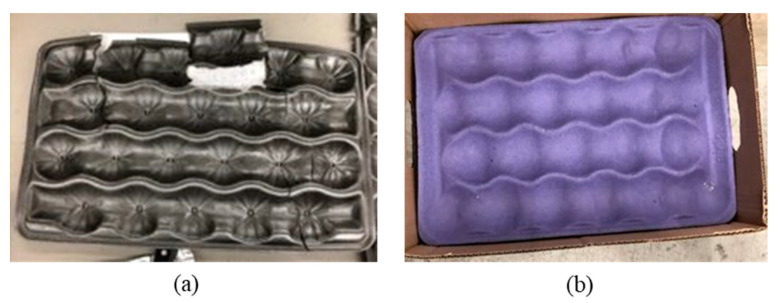
Example of tray damage, (**a**) EPS and (**b**) MF.

For packaging to protect the product, the material must absorb the mechanical shock event to mitigate the event from transferring to the product. After drop testing, the trays were graded based on the level of damage observed using the scale outlined in Section 2.5. The MF trays from the 30 cm and 50 cm drops were graded as good condition. Regardless of drop height, the MF trays showed occasional small tears (<25 cm) and minor creasing. The EPS trays from both the 30 cm and 50 cm drops experience heavy damage, and were graded as poor condition. Figure 11 illustrates the type of damage experienced by the EPS trays as result of the drop testing. This absorption of shock will often damage the packaging materials, especially at refrigerated conditions where the materials, specifically plastics, are more brittle than fiber-based trays [33]. However, although the EPS trays were damaged, the apple bruising was not as prevalent as the MF tray material. This indicates that the EPS tray material, although more susceptible to fracturing due to the storage conditions, absorbed more impact energy during the drop testing as compared to MF. For all of the drop tests, both with the MF and EPS trays, no damage was observed to any of the corrugated containers used. 

## 4. Conclusions

Examined in this research project were two packaging tray materials, molded fiber and expanded polystyrene, commonly used to transport whole apples. To evaluate the protective nature of these trays, packaged product systems were subjected to mechanical drops by free-fall, promoting bruise damage and reducing fruit quality. This study indicates the EPS trays decreased the bruise susceptibility of whole apples compared to the MF trays, regardless of drop height. This was confirmed in both bruise analysis performed on the individual apples as well as comparing the impact accelerations of the apples packaged in these two configurations. The apples packaged using EPS trays experienced significantly less impact acceleration as those packaged inside MF trays, indicating the EPS trays mitigated the shock, thus reducing the severity and level of bruising to the apples. Fruit damage was more prevalent to the middle layers of the packages, noting additional cushioning material may be desirable to reduce bruising in those areas. Based on the data from this study, although both materials prevent direct fruit-to-fruit contact during handling, they do not provide the same level of performance in reducing bruise damage. Therefore, it is imperative to select a packaging material, which can decrease the likelihood of bruising to the apple as a result of mechanical forces experienced during transport and handling. Results from this study would be of great benefit to apple growers and packaging engineers who are seeking to reduce or minimize the effects of bruising to apples during transport.

## Figures and Tables

**Table 1:** Equivalent impact energy (J) of the package types.

Tray Type	Height 30 cm	Height 50 cm
MF	55.36	92.26
EPS	53.89	89.81

## Data Availability

The data presented in this study are available on request from the corresponding author.

## References

[1] Fadiji T., Coetzee C., Pathare P., Opara U.L. (2016). Susceptibility to impact damage of apples inside ventilated corrugated paperboard packages: Effects of package design. Postharvest Biol. Technol..

[2] Opara L.U. (2007). Bruise susceptibilities of “Gala” apples as affected by orchard management practices and harvest date. Postharvest Biol. Technol..

[3] Mukama M., Ambaw A., Opara U.L. (2020). Advances in design and performance evaluation of fresh fruit ventilated distribution packaging: A review. Food Packag. Shelf Life.

[4] Lewis R., Yoxall A., Marshall M.B., Canty L.A. (2008). Characterising pressure and bruising in apple fruit. Wear.

[5] Jarimopas B., Singh S.P., Sayasoonthorn S., Singh J. (2007). Comparison of package cushioning materials to protect post-harvest impact damage to apples. Packag. Technol. Sci..

[6] Stopa R., Szyjewicz D., Komarnicki P., Kuta Ł. (2018). Determining the resistance to mechanical damage of apples under impact loads. Postharvest Biol. Technol..

[7] Pang D.W., Studman C.J., Banks N.H. (1994). Apple bruising thresholds for an instrumented sphere. Trans. ASAE USA.

[8] Bollen A.F., Cox N.R., Dela Rue B.T., Painter D.J. (2001). PH—Postharvest Technology: A descriptor for damage susceptibility of a population of produce. J. Agric. Eng. Res..

[9] Peggie: Technical Problems in the Retail Marketing. https://scholar.google.com/scholar_lookup?title=Technical%20problems%20in%20the%20retail%20marketing%20of%20fruit%20and%20vegetables&publication_year=1968&author=I.%20Peggie.

[10] Wagner T.P. (2020). Policy instruments to reduce consumption of expanded polystyrene food service ware in the USA. Detritus.

[11] Winans K., Marvinney E., Gillman A., Spang E. (2020). An evaluation of on-farm food loss accounting in Life-Cycle Assessment (LCA) of Four California Specialty Crops. Front. Sustain. Food Syst..

[12] Fu H., He L., Ma S., Karkee M., Chen D., Zhang Q., Wang S. (2016). Bruise responses of apple-to-apple impact. IFAC-Pap..

[13] Van Zeebroeck M., Van linden V., Darius P., De Ketelaere B., Ramon H., Tijskens E. (2007). The effect of fruit factors on the bruise susceptibility of apples. Postharvest Biol. Technol..

[14] Zhu Q., Guan J., Huang M., Lu R., Mendoza F. (2016). Predicting bruise susceptibility of “golden delicious” apples using hyperspectral scattering technique. Postharvest Biol. Technol..

[15] Stropek Z., Gołacki K. (2013). The effect of drop height on bruising of selected Apple Varieties. Postharvest Biol. Technol..

[16] Singh S.P., Burgess G., Xu M. (1992). Bruising of apples in four different packages using simulated truck vibration. Packag. Technol. Sci..

[17] Singh S.P., Xu M. (1993). Bruising in apples as a function of truck vibration and packaging. Appl. Eng. Agric..

[18] Peleg K. (1985). Biomechanics of fruits and vegetables. J. Biomech..

[19] Batt G.S., Lussier M., Cooksey K., Northcutt J. (2019). Transportation, handling, and microbial growth performance of molded fiber and expanded polystyrene apple trays. Packag. Technol. Sci..

[20] Ambaw A., Fadiji T., Opara U.L. (2021). Thermo-mechanical analysis in the fresh fruit cold chain: A review on recent advances. Foods.

[21] Fadiji T., Coetzee C.J., Berry T.M., Opara U.L. (2019). Investigating the role of geometrical configurations of ventilated fresh produce packaging to improve the mechanical strength—Experimental and numerical approaches. Food Packag. Shelf Life.

[22] Fadiji T., Coetzee C.J., Opara U.L. (2019). Analysis of the creep behaviour of ventilated corrugated paperboard packaging for handling fresh produce—An experimental study. Food Bioprod. Process..

[23] Fadiji T., Coetzee C.J., Opara U.L. (2020). Evaluating the displacement field of paperboard packages subjected to compression loading using Digital Image Correlation (DIC). Food Bioprod. Process..

[24] Bollen A.F., Nguyen H.X., Dela Rue B.T. (1999). Comparison of methods for estimating the bruise volume of apples. J. Agric. Eng. Res..

[25] Lu F., Ishikawa Y., Kitazawa H., Satake T. (2010). Measurement of impact pressure and bruising of apple fruit using pressure-sensitive film technique. J. Food Eng..

[26] Opara U.L., Pathare P.B. (2014). Bruise damage measurement and analysis of fresh horticultural produce—A review. Postharvest Biol. Technol..

[27] Schoorl D., Holt J.E. (1982). Impact bruising in 3 apple pack arrangements. J. Agric. Eng. Res..

[28] Holt J.E., Schoorl D. (1984). Package protection and energy dissipation in apple packs. Sci. Hortic..

[29] Zarifneshat S., Ghassemzadeh H.R., Sadeghi M., Abbaspour-Fard M.H., Ahmadi E., Javadi A., Shervani-Tabar M.T. (2010). Effect of impact level and fruit properties on golden delicious apple bruising. Am. J. Agric. Biol. Sci..

[30] Ahmadi E. (2012). Bruise susceptibilities of kiwifruit as affected by impact and fruit properties. Res. Agric. Eng..

[31] Ahmadi E., Ghassemzadeh H.R., Sadeghi M., Moghaddam M., Neshat S.Z. (2010). The effect of impact and fruit properties on the bruising of peach. J. Food Eng..

[32] Van Zeebroeck M., Van linden V., Ramon H., De Baerdemaeker J., Nicolaï B.M., Tijskens E. (2007). Impact damage of apples during transport and handling. Postharvest Biol. Technol..

[33] Marsh K., Bugusu B. (2007). Food packaging—Roles, materials, and environmental issues. J. Food Sci..

